# 801. IDSA Featured Oral Abstract: 25 Years of Varicella Vaccination Program in the United States: Health Impact during 1995–2019

**DOI:** 10.1093/ofid/ofac492.060

**Published:** 2022-12-15

**Authors:** Mona Marin, Jessica W Leung, Adriana S Lopez, Michael Melgar, Tara C Anderson, Aaron T Curns, Kathleen L Dooling

**Affiliations:** Centers for Disease Control and Prevention, Atlanta, Georgia; Centers for Disease Control and Prevention, Atlanta, Georgia; Centers for Disease Control, Atlanta, Georgia; Centers for Disease Control and Prevention, Atlanta, Georgia; Centers for Disease Control and Prevention, Atlanta, Georgia; Centers for Disease Control and Prevention, Atlanta, Georgia; Centers for Disease Control and Prevention, Atlanta, Georgia

## Abstract

**Background:**

In 1995, the United States was the first country to introduce universal childhood varicella vaccination. High vaccine coverage was attained among young children, ≥ 90% since 2007. In 2007, the policy was changed from 1-dose to a 2-dose program. We describe the impact of 25 years of the U.S. varicella vaccination program on varicella disease nationally.

**Methods:**

We reviewed published data and analyzed overall and age-specific trends for rates from the pre-vaccine period (1990–1994) through 2019 for varicella incidence using National Notifiable Diseases Surveillance System data, hospitalizations using National Inpatient Sample data, and deaths using National Center for Health Statistics data. We present trends in persons aged < 50 years, which captures most varicella burden and avoids misclassified herpes zoster in older people. Outbreak (≥ 5 varicella cases epidemiologically linked) characteristics were assessed for 1995–2019 and were informed by published data and analysis of surveillance data reported to CDC.

**Results:**

Within the 10 years of the 1-dose program, varicella incidence, hospitalization, and mortality rates declined dramatically (71%–90%) vs. pre-vaccine. However, limited transmission continued in school settings which informed the change to a 2-dose policy. By 2019, declines reached > 97% for incidence and 94% and 97% for hospitalizations and deaths, respectively. The greatest decline occurred among persons aged < 20 years, born during the varicella vaccination program, with 99%, 97%, and > 99% reduction in incidence, hospitalizations, and deaths, respectively. The 2-dose program further reduced the number, size, and duration of outbreaks vs. the 1-dose program; over the entire program, the proportion of outbreaks with < 10 cases increased from 28% to 73%.

**Conclusion:**

The varicella vaccination program significantly reduced varicella morbidity and mortality in the U.S. Twenty-five years into the program, pediatric varicella hospitalization has become a rare event and varicella deaths in persons aged < 20 years are practically eliminated in the U.S. Annually, > 3.8 million cases, 10,500 hospitalizations, and 100 deaths from varicella are now prevented in the United States due to the varicella vaccination program.

**Disclosures:**

**All Authors**: No reported disclosures.

